# A study of ribonuclease activity in venom of vietnam cobra

**DOI:** 10.1186/s40781-017-0145-5

**Published:** 2017-09-25

**Authors:** Thiet Van Nguyen, A. V. Osipov

**Affiliations:** 1 Institute of Biotechnology, Vietnam Academy of Science and Technology (VAST), Hanoi, Vietnam; 2 Shemyakin-Ovchinnikov Institute of Bioorganic Chemistry, Russian Academy of Sciences (RAS), Moscow, Russia

**Keywords:** Acidic optimum pH, Conformational stability, Gel filtration, Ionic strength, Isoforms, Thermostability, Venom RNase, Vietnam cobra

## Abstract

**Background:**

Ribonuclease (RNase) is one of the few toxic proteins that are present constantly in snake venoms of all types. However, to date this RNase is still poorly studied in comparison not only with other toxic proteins of snake venom, but also with the enzymes of RNase group. The objective of this paper was to investigate some properties of RNase from venom of Vietnam cobra *Naja atra*.

**Methods:**

Kinetic methods and gel filtration chromatography were used to investigate RNase from venom of Vietnam cobra.

**Results:**

RNase from venom of Vietnam cobra *Naja atra* has some characteristic properties. This RNase is a thermostable enzyme and has high conformational stability. This is the only acidic enzyme of the RNase A superfamily exhibiting a high catalytic activity in the pH range of 1–4, with pH_opt_ = 2.58 ± 0.35. Its activity is considerably reduced with increasing ionic strength of reaction mixture. Venom proteins are separated by gel filtration into four peaks with ribonucleolytic activity, which is abnormally distributed among the isoforms: only a small part of the RNase activity is present in fractions of proteins with molecular weights of 12–15 kDa and more than 30 kDa, but most of the enzyme activity is detected in fractions of polypeptides, having molecular weights of less than 9 kDa, that is unexpected.

**Conclusions:**

RNase from the venom of Vietnam cobra is a unique member of RNase A superfamily according to its acidic optimum pH (pH_opt_ = 2.58 ± 0.35) and extremely low molecular weights of its major isoforms (approximately 8.95 kDa for RNase III and 5.93 kDa for RNase IV).

## Background

Since ancient times snake venom has been considered as the precious medicinal source. Not by chance that the symbol of medicine in all over the world is the image of a snake releasing venom. Today we know that snake venom is the source, very rich in biologically active substances, such as toxins, proteins, enzymes and toxic peptides. Snake venom affects many organs and organ systems in the body such as the nervous system, blood and cardiovascular system, respiratory and muscular systems... Snake venom is used to treat various diseases, including cardiovascular diseases [1], hypertension [2] and osteoarthritis [3]. The venom of some snakes showed anti-cancer effects and is used to develop anti-cancer drugs [4–7].

Snake venom is a biological fluid having the highest RNase activity [8]. RNase is one of the toxic enzymes that are permanently present in snake venoms of all kinds [9, 10]. However, up to now snake venom RNase is little studied in comparison not only with the enzymes of RNase group, but also with the other proteins of snake venoms. Probably this is the reason that so far we still do not understand the role of this enzyme in the toxic action of the snake venoms in general.

Moreover, snake venom RNase is a secreted enzyme, so this RNase belongs to the RNase A superfamily. At present has been known that the RNase A superfamily is a large family of secreted RNases in vertebrates from amphibians to mammals, including species *Homo sapiens* with RNase A (an enzyme from bovine pancreas) as a prototype [11, 12]. In the last time the main function of the enzymes of RNase A superfamily is thought to be involved in the host defense system. Many members of this superfamily possess antimicrobial [13–19], antiviral [20–23], cytotoxic [24–29] and other biological actions [26, 30–32], as observed for the most of RNases of the RNase A superfamily in human [33–35] and for RNases of this superfamily in other organisms [13, 19, 26, 36, 37]. From this context, we believe that the permanent presence of RNase in snake venoms of all kinds is not accidental; and the fact that snake venom is a biological fluid having very high ribonucleolytic activity may reflect a certain role of this enzyme in the toxic effects of snake venom.

In this work, the results of studying some properties of RNase from the venom of Vietnam cobra will be presented. Information about these properties may help to understand the role of RNase in general toxic effects of snake venom.

## Methods

### Materials

The dry (lyophilized) venom of cobra the *Naja atra* (purchased from Vinh Son Snake farming village, Vinh Tuong District, Vinh Phuc Province) was used as a source of RNase. Total RNA from the yeast *Torula* (purchased from Sigma) was used as a substrate for the determination of RNase activity. Glycine from Prolabo, sodium citrate, citric acid, sodium chloride, sodium hydroxide, hydrochloric acid and other chemicals are analytically pure.

The initial enzyme solution (E_0_) was prepared by dissolving 2–10 mg or more of snake venom in the appropriate buffer solution, and then centrifuged at 10000 rpm for 10 min to remove the insoluble material.

### Methods

#### Heat treatment

To study the thermostability of the RNase activity in Vietnam cobra venom, several series of enzyme samples with concentrations of 2–10 mg/ml of lyophilized venom were heating in a water bath at different temperatures from 30 to 100 °C over in the same period of time (5 min). After thermal treatment, the enzyme samples were cooled on ice and centrifuged at 10.000 rpm for 10 min to remove the residue of inactivated proteins and then RNase activity in the supernatant liquids was determined, as described below.

#### Determination of the optimum pH

For studying the influence of pH on RNase activity and determining the optimum pH (pH_opt_) of the enzyme, RNA hydrolysis reactions were carried out in 10 mM Phosphate buffer (P-buffer) at pH 5.8–8.0, in 10 mM Glycine buffer (G-buffer) at pH 1.5–4.0 and in 10 mM mixed buffer (M-buffer) at pH 1.0 to 9.5, prepared in the equal molar ratio (1: 1: 1: 1) from glycine, citrate, phosphate and tris-HCl.

#### Study the effect of ionic strength

The influence of ionic strength on RNase activity was studied by performing RNA hydrolysis at different concentrations of G- and M-buffers (from 5 to 100 mM), NaCl and (NH_4_)_2_SO_4_ (from 0 to 100 mM), MgSO_4_ (from 0 to 4 mM) and EDTA (from 0 to 10 mM).

#### Treatment with trypsin

Venom RNase in the enzyme preparation E_0_ was incubated with trypsin (at a ratio trуpsin/protein in preparation E_0_ equal to 1/13) at 25 °C, and then the RNase activity remaining in the reaction mixture was determined at various time intervals.

#### Gel-filtration chromatography

140 mg of the dried venom of Vietnam cobra *Naja atra* were separated by a gel-filtration column (ø2.5 × 90 cm) with Sephadex G50sf as a carrier in 0.1 M ammonium acetate buffer pH 6.2 at a flow rate of 15 ml/h, the volume of collected fractions was 5 ml. In the case of gel-filtration on a Superdex 75 column (ø0.9 × 60 cm), different amounts of cobra venom (10-50 mg) were used for fractionation, and the chromatographic process was carried out in 10 mM citrate buffer pH 5.25 or in 10 mM phosphate buffer pH 7.4 at a flow rate of 1 ml/min, the volume of collected fractions was 1 ml. After chromatography, the ribonucleolytic activity was determined in all fractions obtained as described below.

#### RNase activity assay

RNA hydrolysis reactions were carried out in 10 mM glycine buffer pH 2.6 at room temperature for 30 s in a total volume of 1 ml of the reaction mixture [8]. The hydrolysis reaction is initiated by adding *x* μl of the enzyme solution in the (1000 - *x*) μl of RNA solution prepared in glycine buffer. RNase activity was evaluated by the increase in OD_260_ of the reaction mixture during the hydrolysis of the RNA substrate. One unit of RNase activity is defined as the amount of enzyme which hydrolyzes RNA causing an increase in OD_260_ value of the reaction mixture by 1 unit.

#### Protein determination

The protein content in the fractions of gel filtration was automatically recorded at a wavelength of 280 nm.

## Results

### Thermostability of the enzyme

**Fig. 1:**
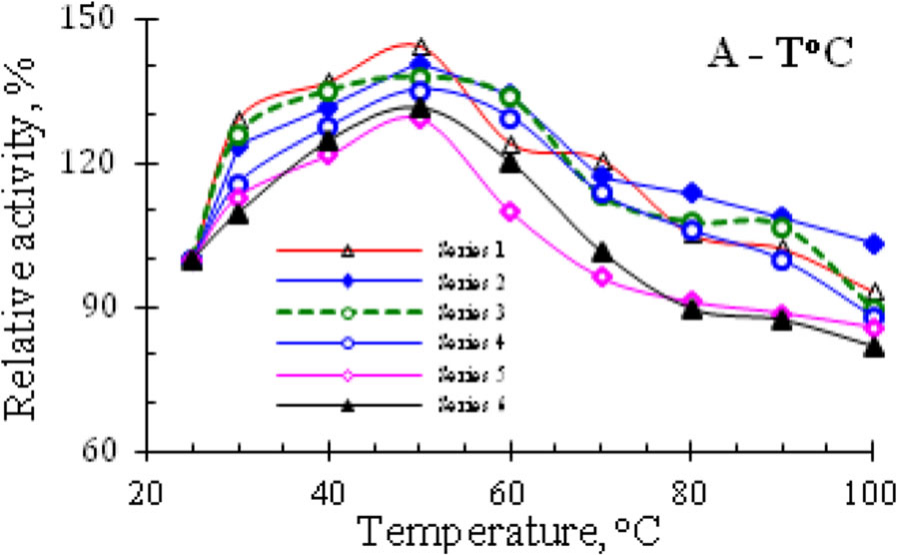
Effect of temperature on the activity of RNase in the venom from Vietnam cobra. Ribonucleolytic activity of enzyme solutions E_0_ of six series with different concentrations of snake venom was determined after their 5-min heating in a water bath at different temperatures. The activity of initial enzyme solutions E_0_ before heating was considered to be 100%

In experiments studying the thermostability of RNase activity in venom of Vietnam cobra, the enzyme samples were heat-treated as described in the section Methods. The results of the determinination of ribonucleolytic activity remained in the supernatant liquids after centrifugation of several series of heat-treated enzyme samples, are summarized in Table 1, and the curves, illustrating the temperature dependence of the relative activity of RNase, are shown in Fig. 1.

**Table 1:** Results of determination of ribonucleolytic activity (A, OD_260_) remained in the supernatant liquids after centrifugation of the heat-treated enzyme samples

T^o^C	Ribonucleolytic activity remained after heat treatment											
	Series 1		Series 2		Series 3		Series 4		Series 5		Series 6	
	OD_260_	%	OD_260_	%	OD_260_	%	OD_260_	%	OD_260_	%	OD_260_	%
25	0.165	100.0	0.206	100.0	0.190	100.0	0.166	100.0	0.229	100.0	0.228	100.0
30	0.214	129.3	0.254	123.6	0.239	125.9	0.193	115.9	0.259	113.0	0.250	109.5
40	0.227	137.0	0.270	131.6	0.257	135.0	0.212	127.4	0.279	121.7	0.284	124.6
50	0.239	144.5	0.289	140.7	0.262	137.9	0.224	135.2	0.297	129.2	0.299	131.4
60	0.206	124.2	0.276	134.3	0.254	133.5	0.215	129.4	0.252	109.8	0.274	120.1
70	0.200	121.0	0.241	117.2	0.215	113.2	0.189	113.8	0.221	96.1	0.231	101.4
80	0.174	105.3	0.234	113.6	0.205	107.6	0.176	105.9	0.209	91.0	0.204	89.4
90	0.169	102.4	0.223	108.7	0.203	106.7	0.166	99.9	0.203	88.7	0.198	87.1
100	0.155	93.5	0.212	103.2	0.170	89.4	0.146	88.1	0.197	85.7	0.186	81.6

The data in Table 1 and the curves in Fig. 1 showed that RNase activity in venom of Vietnam cobra *N. atra* is highly thermostable. It has the maximum activity after heating the enzyme solution at 50 °C for 5 min and in this case the RNase activity was increased by about 29–45%. Moreover, after boiling for 5 min in a water bath this enzyme retained approximately 82–103% of its original activity.

### Effect of pH on the enzyme activity

**Fig. 2:**
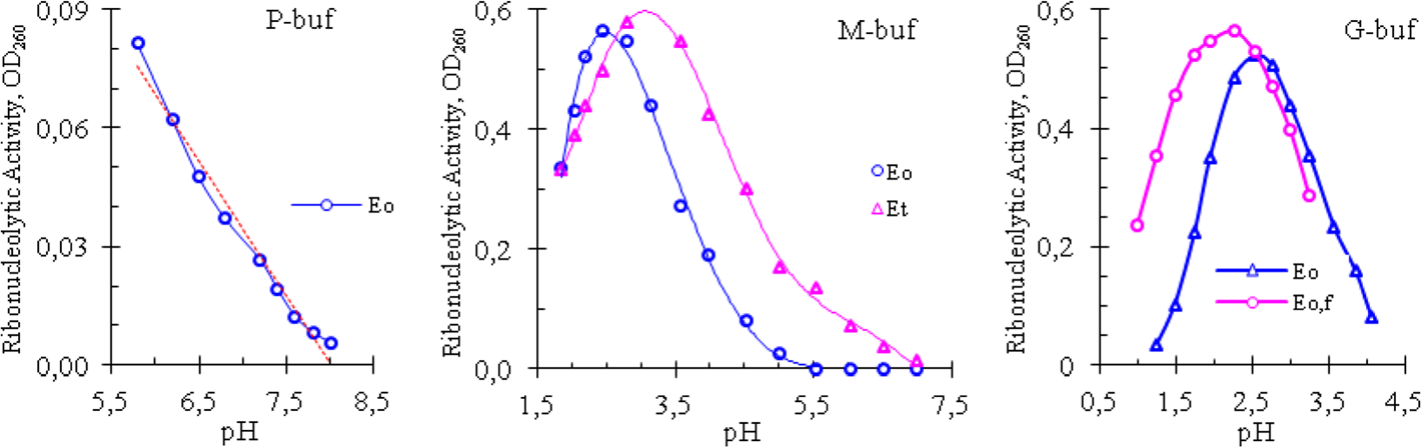
pH-dependence of ribonucleolytic activity of cobra venom. P-buf – Phosphate buffer; M-buf – mixed buffer; G-buf – Glycine buffer; E_0_ – The initial enzyme solution, prepared from lyophilized venom; E_0,f_ – The initial enzyme solution, prepared from fresh venom; E_t_ – The heat-treated (boiled in the water bath for 5 min) enzyme solution E_0_

The effect of pH on the cobra venom RNase was studied by measuring its enzymatic activity at different pH values of phosphate, glycine, and mixed buffers. The results of these experiments are shown in Fig. 2.

The curves in Fig. 2 showed that the venom RNase of Vietnam cobra had a high catalytic activity in an acid medium: its activity was high in the pH range from 1.5 to 4.0 in both G- and M-buffers, and was dropped almost linearly in P-buffer when pH increased from 5.8 to 8.0. As can be seen in Fig. 2, and according to the results obtained, the optimum pH (pH_opt_) for RNase activity of Vietnam cobra venom varied in a rather wide range: pH_opt_ = 2.58 ± 0.35.

### Effects of ionic strength

**Fig. 3:**
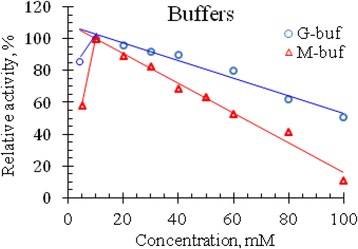
Effect of buffer concentration on RNase activity in the venom of Vietnam cobra. M-buf – mixed buffer; G-buf – Glycine buffer

In the previous section it was shown that venom RNase of Vietnam cobra exhibits the highest activity in an acidic medium, so the effect of ionic strength on the activity of this RNase was studied in the pH range from 1.5 to 4.0. The results of the experiment for determining the activity of RNase at different concentrations of G- and M-buffers, are summarized in Table 2 and are illustrated in Fig. 3.

**Table 2:** RNase activity of Vietnam cobra venom at different concentrations of buffers

Buffer	Activity	Buffer’s concentrations, mM									
		4	5	10	20	30	40	50	60	80	100
M-buf	OD_260_	-	0.265	0.456	0.407	0.375	0.313	0.288	0.239	0.190	0.048
	%	-	58.16	100.0	89.34	82.28	68.68	63.25	52.41	41.58	10.48
G-buf	OD_260_	0.506	-	0.588	0.564	0.540	0.530	-	0.470	0.366	0.300
	%	86.10	-	100.0	95.92	91.84	90.14	-	79.93	62.24	51.02

As seen in Fig. 3, RNase activity in Vietnam cobra venom is strongly influenced by ionic strength. The enzyme had the highest activity at 10 mM concentration of both G- and M-buffers. In the range of buffer concentrations from 10 to 100 mM, venom RNase activity is almost linearly decreased with increasing buffer concentration. Moreover, at 100 mM concentration of these buffers, the enzyme retained 10.5% and 51% of its activity, respectively (i.e., the enzyme activity was 10.5 and 51% of its maximum activity in the corresponding buffers).

**Fig. 4:**
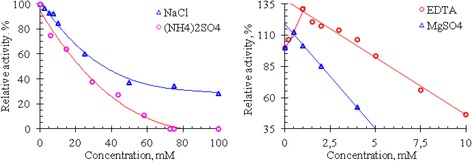
Effect of salts and EDTA on the activity of RNase in the venom of Vietnam cobra

On the other hand, It is known that NaCl and (NH_4_)_2_SO_4_ are the salts most commonly used in isolation and purification of enzymes and many enzymes require divalent metal ions for their catalysis. Therefore, the effects of these salts, EDTA and MgSO_4_ on the RNase activity of Vietnam cobra venom are assessed by measuring the enzyme activity in the presence of these substances. The results of these experiments are shown in Fig. 4.

As seen in Fig. 4, cobra venom RNase activity is strongly influenced by NaCl and (NH_4_)_2_SO_4_: the activity of this enzyme is rapidly reduced with increasing salt concentration. At 100 mM concentration of NaCl, the RNase activity is only 28% compared to its activity in the absence of this salt, whereas at ammonium sulfate concentration higher 70 mM the venom RNase lost completely its activity. In addition, the activity of this RNase is also considerably reduced in the presence of MgSO_4_ and EDTA. Venom RNase activity is remained only about 52.6% at 4 mM concentration of MgSO_4_ and 46.7% at 10 mM concentration of EDTA. These data show that Mg^2+^ ions are not essential for the catalytic activity of RNase from Vietnam cobra venom.

### Susceptibility of RNase from Vietnam cobra venom to the proteolytic action of trypsin

**Fig. 5:**
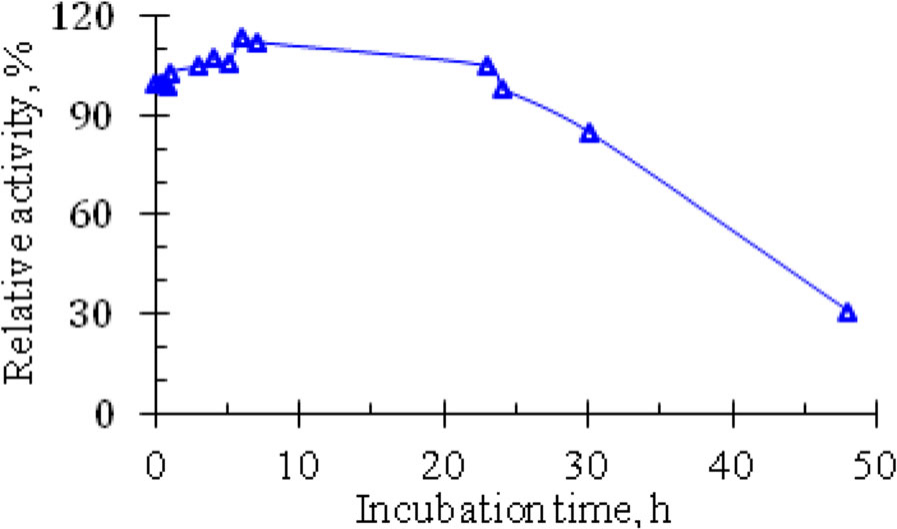
Effect of the proteolytic action of trypsin on RNase in the venom of Vietnam cobra

Conformational stability is a very important parameter determining the biological activity of proteins in general and enzymes in particular. In addition to thermal stability, another expression of the conformational stability of protein molecules is their susceptibility to proteolytic action of proteinases – hydrolytic enzymes present in all cells. A curve illustrating the effect of the proteolytic action of trypsin on the RNase from Vietnam cobra venom is shown in Fig. 5.

The curve in Fig. 5 showed that the venom RNase activity is not reduced, but even increased when the crude enzyme solution E_0_ is incubated with trypsin for one day. The enzyme activity began to decrease after a 24-h incubation and RNase still retained about 30% of its activity after 48 h of incubation with trypsin. These data indicate that RNase from the venom of Vietnam cobra is very resistant to the proteolytic action of trypsin. This means that RNase from Vietnam cobra venom has a very high conformational stability.

### Multiple molecular forms and abnormal distribution of their activity

**Fig. 6:**
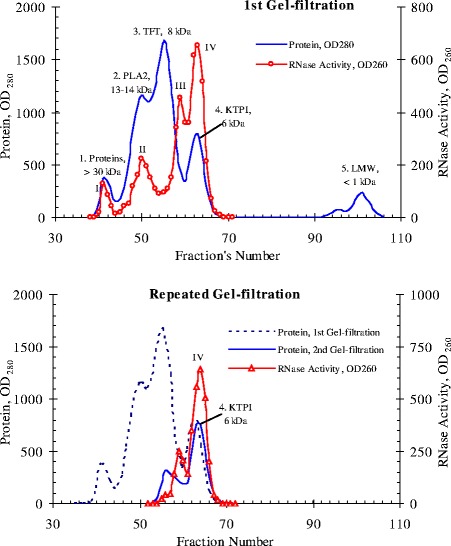
The elution profiles of proteins of Vietnam cobra venom at gel filtration on a Sephadex G50sf column. The first gel filtration was carried out with the whole cobra venom (*top*) and repeated gel filtration of peak 4 from the first gel filtration (*bottom*). KTPI – Kunitz-type proteinase inhibitors, LMW – *Low* molecular weight substances, PLA2 –Phospholipase A2, TFT – Three-finger toxins

According to our preliminary data, RNase activity in the venom of Vietnam cobra *N. atra* is abnormally high [8]. One of the objectives of this work is to find the factors behind this phenomenon. To this end, the proteins of the dried venom of Vietnam cobra are fractionated by gel filtration on a Sephadex G50sf column (ø2.5 × 90 cm). The results of this experiment are shown in Fig. 6.

As shown in Fig. 6, when fractionated by gel filtration on a Sephadex G50sf column, proteins of venom from Vietnam cobra are divided into four conventional protein peaks (1–4) and one peak of low-molecular substances (LMW), as expected.

Peak 1 consists of components of the venom with molecular weights above 30 kDa (e.g., trimeric phospholipase A2, PLA2) and contains traces of RNase activity. Peak 2 corresponds to fractions of PLA2 and has, as expected, a moderate RNase activity. However, the bulk of the RNase activity is concentrated in peak 4 corresponding to fractions of Kunitz-type proteinase inhibitors (KTPI), and a much smaller portion of the activity is localized in the tail of peak 3, which is the biggest protein peak and corresponds to fractions of three-finger toxins (TFT, including alpha-neurotoxins and cytotoxins). This distribution of RNase activity in gel filtration fractions is fundamentally contrary to the available data in the literature [9, 38], because according to these data, RNase of snake venom has a molecular weight of 12–15 kDa and should be contained in the same fractions as PLA2.

**Fig. 7:**
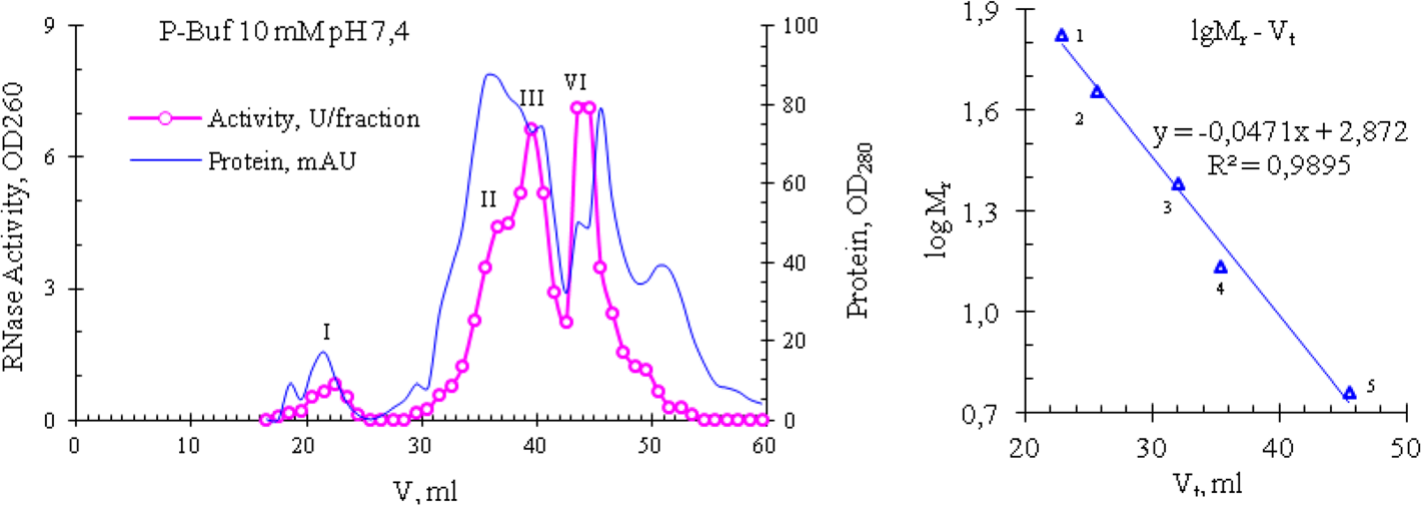
The elution profile of proteins of Vietnam cobra venom at gel filtration on a Superdex 75 column (*left*) and calibration curve for determining molecular weights of the enzyme (*right*). Standard proteins: 1. Bovine serum albumin (BSA, 67 kDa); 2. Ovalbumin (45 kDa); 3. Trypsin (24 kDa); 4. RNase A (13.7 kDa); 5. Insulin (5.8 kDa). Conditions of gel filtration: 10 mM phosphate buffer pH 7.4; flow rate 1 ml/min

Later we performed a repeated gel filtration of peak 4 under the same conditions and found that its own RNase activity is not scattered on peaks under these chromatographic conditions, and is again present in fractions corresponding to Kunitz inhibitors (i.e., at peak 4), and partly - in the tail of peak 3 (Fig. 6, bottom). Thus, these data can allow suggesting that the abnormally high RNase activity of venom from Vietnam cobra is possibly due to a new type RNase, observed in fractions of Kunitz inhibitors with molecular weight of 6 kDa. Gel Filtration on a Superdex 75 column (ø0.9 × 60 cm) also gave similar results: RNase from Vietnam cobra venom is separated into 4 isoforms which differed in molecular size (Fig. 7).

These isoforms are denoted as RNase I, II, III and IV, their molecular weights respectively are >30; 12.94 ± 1.74 (*n* = 10); 8.95 ± 1.34 (*n* = 18) and 5.93 ± 0.38 kDa (*n* = 18), which were determined from a calibration curve created with using bovine serum albumin (BSA), ovalbumin, trypsin, RNase A and insulin as the standard proteins. Note: Sometimes the high molecular weight isoform RNase I is absent and isoform RNase II is often appeared as the left shoulder of isoform RNase III, whereas isoforms RNase III and RNase IV are the main forms and in some experiments we have obtained only these two isoforms. In the experiment presented in Fig. 7, the ribonucleolytic activity of the isoforms RNase I, II, III and IV has the following distribution: 4.5, 19.2, 35.8 and 40.5%, respectively. In general the results of the separation of snake venom RNase on the Superdex 75 column are similar to those that are obtained by separation on the Sephadex G50sf column. These results also reflect the abnomal distribution of RNase activity from the venom of Vietnam cobra.

## Discussion

From the results presented above, we see that RNase in Vietnam cobra venom is a thermostable enzyme. This RNase exhibits the highest catalytic activity after heating enzyme solution for 5 min at 50 °C (this thermal treatment increases the enzyme activity by 30–45%) and its activity is reduced insignificantly or almost does not change when the enzyme solution is boiled in water bath for 5 min. According to the thermostability, RNase from Vietnam cobra venom is similar to all other enzymes of the RNase A superfamily [12], but this enzyme is differed from RNases in the venoms of cobra *Naja oxiana* from Central Asia of former Soviet Union and cobra *Naja naja* from region Guntur in India (RNase from these cobras completely inactivated at 70 °C [39] or showed the highest activity at 40 °C [40], respectively).

If according to thermostability RNase from venom of Vietnam cobra is similar to other enzymes of the RNase A superfamily, but this RNase is differed from all other known members of this superfamily by the dependence on pH of its ribonucleolytic activity: All known until today enzymes of the RNase A superfamily, including RNases from the venoms of Indian cobra *N. naja* [41] and Central Asian cobra *N. oxiana* of the former Soviet Union [42] have pH_opt_ in the pH range of 6 to 8, and mostly with pH_opt_ > 7 [12, 43], whereas RNase from Vietnam cobra venom is highly active in the acidic pH range, with pH_opt_ = 2.58 ± 0.35. Since the optimum pH of RNase from Vietnam cobra venom is very low, therefore, the RNA hydrolysis reaction catalyzed by this enzyme was carried out in glycine buffer (G-buffer) or in mixed buffer (M -buffer) at pH 2.6. Although the enzyme is most active at 10 mM concentration of both buffers, at increasing buffers’ concentration from 10 to 100 mM, the enzyme activity is decreased slowly in G-buffer, but is dropped rapidly in M-buffer, and at buffer concentration of 100 mM, venom RNase retains 51 and 10.5% of its activity in G- and M-buffers, respectively. On the other hand, NaCl and (NH_4_)_2_SO_4_ are the salts often used in the isolation and purification of enzymes, and these salts also significantly decreased the RNase activity in Vietnam cobra venom: In the presence of NaCl at 100 mM concentration venom RNase retains only 28% of activity in comparison to its activity in the absence of this salt, and at ammonium sulfate concentration higher 70 mM the enzyme lost completely its activity. The activity of Vietnam cobra venom RNase is also considerably reduced in the presence of MgSO_4_ and EDTA. The enzyme has the highest activity at 0.5 and 1 mM of MgSO_4_ and EDTA, respectively; and its activity is also almost linearly decreased with increasing concentration of MgSO_4_ (from 0.5 mM to 4 mM) and EDTA (from 1 mM to 10 mM). The activity of venom RNase is retained only about 52.6% at 4 mM concentration of MgSO_4_ in comparison to its activity in the absence of this salt. This effect of Mg ions is completely different from the character of their influence on venom RNase from other sources. In the same concentration range, Mg^2+^ is required for the activity of venom RNase from Indian cobra *N. naja* [40] and increases the activity of venom RNase from cobra *N. oxiana* in Central Asia of the former Soviet Union up to 10 times [39]. Thus, Mg^2+^ ions, obviously, do not play a role in the catalysis of RNase from Vietnam cobra venom. This is confirmed by the fact that the activity of this enzyme is increased by 25% in the presence of EDTA at 1 mM concentration. However, the enzyme activity is decreased at Mg^2+^ ions concentrations higher 1 mM. In our opinion, this decrease in enzyme activity at concentrations of EDTA higher 1 mM, is not associated with the loss of Mg^2+^ ions contained in the molecules of enzyme (if enzyme preparation has a certain amount of Mg^2+^), but is probably due to the effect of EDTA anions on the structure of the enzyme, thereby leading to a decrease in RNase activity.

Today we know that some enzymes of the RNase A superfamily such as RNases from several species of frogs [24, 27, 37] or bovine seminal RNase (BS-RNase) [25, 26], possess cytotoxic activity because these RNases do not interact with ribonuclease inhibitor (RI) in then cytoplasm [44]. These RNases do not interact with RI because of the small size of their molecules (in the case of RNases from frogs) or too large size (in the case of BS-RNase) compared to RNase A and other enzymes of the RNase A superfamily. RNase from frog *R. pipiens* has high anti-cancer activity, acts specifically on tumor cells, but does not affect normal healthy cells, and therefore it is called *onconase*. This RNase has a small size; its polypeptide chain is about ten amino acid residues shorter than that of RNase A due to mutations leading to the loss of amino acids which contact RI, so onconase does not interact with RI [27, 29, 45]. Moreover, some studies have shown that the higher conformational stability of RNase, the higher its cytotoxic activity [46]. A very important feature of RNase from Vietnam cobra venom is its low susceptibility to the proteolytic action of trypsin: the activity of this RNase is not lost, is not reduced, and is even increased slightly during the first hours of incubation of enzyme preparation E_0_ with trypsin at 25 °C (at the ratio of trypsin/protein in the preparation E_0_, equal to 1/13), the catalytic activity of venom RNase started to decline only after 24 h of incubation. The low susceptibility to the proteolytic action of trypsin and high thermostability are reliable indicators of the conformational stability of protein molecules. These parameters show that RNase from Vietnam cobra venom has very high conformational stability. On the other hand, according to the results of gel filtration chromatography, the main isoforms of RNase (RNase III and RNase IV) from Vietnam cobra venom have very low molecular weights, so they can not interact with RI. These data allow supposing that two low molecular weight isoforms of RNase from Vietnam cobra venom may have cytotoxic activity. This will be the objective of our research in the future.

## Conclusions

From the obtained results presented above, it can be concluded as follows:

1. RNase from Vietnam cobra venom has the highest catalytic activity in the acidic pH range, with pH_opt_ = 2.58 ± 0.35.
2. This RNase has very high conformational stability, which manifests itself in its thermostability and low susceptibility to the proteolytic action of trypsin.
3. The enzyme does not need ions Mg^2+^ for catalysis. Its activity is considerably reduced due to increasing the ionic strength (concentration of buffer or salts) in the reaction mixture.
4. This RNase has 4 isoforms (RNase I, II, III and IV), with molecular weights, respectively, of more than 30, 12.94, 8.95 and 5.93 kDa, of which two low molecular weight RNases III and IV are the main isoforms.

## Abbreviations

BSA: Bovine serum albumin
BS-RNase: Bovine seminal ribonuclease
E_0_: Enzyme solution prepared from lyophilized venom
E_0, f_: Enzyme solution prepared from fresh venom
E_t_: The heat-treated (boiled in the water bath for 5 min) enzyme solution E_0_
G-buffer: Glycine buffer
KTPI: Kunitz-type proteinase inhibitors
LMW: Low molecular weight substances
M-buffer: Mixed buffer
P-buffer: Phosphate buffer
pH_opt_: Optimum pH
PLA2: Phospholipase A2
RI: Ribonuclease inhibitor
RNase A: Bovine pancreatic ribonuclease
RNase: Ribonuclease
TFT: Three-finger toxins
